# IL-1 Receptor Antagonist Treatment Aggravates Staphylococcal Septic Arthritis and Sepsis in Mice

**DOI:** 10.1371/journal.pone.0131645

**Published:** 2015-07-02

**Authors:** Abukar Ali, Manli Na, Mattias N. D. Svensson, Malin Magnusson, Amanda Welin, Jan-Christoph Schwarze, Majd Mohammad, Elisabet Josefsson, Rille Pullerits, Tao Jin

**Affiliations:** Department of Rheumatology and Inflammation Research, Institution of Medicine, The Sahlgrenska Academy at University of Gothenburg, Gothenburg, Sweden; Harvard Medical School, UNITED STATES

## Abstract

**Background:**

Interleukin-1 receptor antagonist (IL-1Ra) is the primary therapy against autoinflammatory syndromes with robust efficacy in reducing systemic inflammation and associated organ injury. However, patients receiving IL-1Ra might be at increased risk of acquiring serious infections.

**Aims:**

To study whether IL-1Ra treatment deteriorates *Staphylococcus aureus* (*S*. *aureus*) septic arthritis and sepsis in mice.

**Method:**

NMRI mice were treated with anakinra (IL-1Ra) daily for 7 days before intravenous inoculation with *S*. *aureus* strain Newman in both arthritogenic and lethal doses. The clinical course of septic arthritis, histopathological and radiological changes of the joints, as well as the mortality were compared between IL-1Ra treated and control groups.

**Results:**

IL-1Ra treated mice developed more frequent and severe clinical septic arthritis. Also, the frequency of polyarthritis was significantly higher in the mice receiving IL-1Ra therapy. In line with the data from clinical arthritis, both histological and radiological signs of septic arthritis were more pronounced in IL-1Ra treated group compared to controls. Importantly, the mortality of IL-1Ra treated mice was significantly higher than PBS treated controls.

**Conclusion:**

IL-1Ra treatment significantly aggravated *S*. *aureus* induced septic arthritis and increased the mortality in these mice.

## Introduction

Anakinra, a recombinant human interleukin-1 receptor antagonist (IL-1Ra), that inhibits the binding of IL-1 to its receptors, has displayed tremendous efficacy on controlling disease flares and inflammation-mediated organ damage in autoinflammatory syndromes [1, 2]. Autoinflammatory syndromes are a broad range of diseases that are characterized by chronic and recurrent attacks of fever and multisystemic inflammation. In contrast to autoimmune diseases, where the disease is mostly caused by abnormal function of the adaptive immune system, patients with autoinflammatory disorders do not produce antigen-specific T cells, B cells and autoantibodies. Instead, the autoinflammarory syndromes are characterized by abnormal function of the innate immune system. The majority of autoinflammatory syndromes are caused by inappropriate activation of the inflammasome, a component of the innate immune system, consequently leading to an abnormal over-secretion of the pro-inflammatory cytokine IL-1 [2–4]. Besides autoinflammatory syndromes, IL-1Ra has also been shown to be effective for treatment of certain immune dysregulatory conditions [5–8], and a more prevelant joint disease—gout [9, 10].

The other side of the coin is that anti-cytokine therapies are usually associated with an increased overall risk of infections [11] [12]. In mouse experiments, IL-1 receptor deficiency led to more severe *S*. *aureus* septic arthritis and higher mortality in staphylococcal sepsis compared to wild type animals [13]. In humans, metaanalyses demonstrated that high doses of anakinra increase the risk of serious infections, especially when patients have comorbidity factors [14]. However, it remains unknown whether IL-1Ra treatment increases the risk for a particular infection e.g. septic arthritis. Septic arthritis remains one of the most dangerous joint diseases due to its rapidly progressive disease character. The mortality rate of patients with septic arthritis is high (10–25%) and up to 50% of patients develop permanent joint dysfunction [15, 16]. *Staphylococcus aureus* (*S*. *aureus*) is the main cause of septic arthritis and risk factors include immunosuppression, increased age as well as preexisting joint disease and diabetes mellitus [17].

In present study, we investigated the susceptibility of mice receiving IL-1Ra to *S*. *aureus* septic arthritis and sepsis in our well-established mouse model. The data demonstrate that IL-1Ra treatment significantly aggravates septic arthritis and increases the mortality in mice.

## Materials and Methods

### Mice

Female NMRI mice, 6–8 weeks old, were purchased from Charles River Laboratories (Sulzfeld, Germany). They were bred and housed in the animal facility of the Department of Rheumatology and Inflammation Research, University of Gothenburg. They were kept under standard conditions of temperature and light, and were fed laboratory chow and water *ad libitum*. The ethical committee of animal research of Gothenburg approved the study.

### *S*. *aureus* preparation

For induction of stpahylococcal septic arthritis and sepsis in mice, *S*. *aureus* strain Newman were used and prepared as previously described [18]. For phagocytosis assays, *S*. *aureus* strain RN4220 harboring the pCN-GFP plasmid for constitutive expression of green fluorescent protein (GFP) (a kind gift from Dr. Maria Lerm, Linköping University, Sweden) was used[19]. Before experiments, the bacterial solution was thawed, washed in PBS, and adjusted to the concentration required.

### Experimental protocols for staphylococcal septic arthritis and sepsis

Five separate *in vivo* experiments were performed for the staphylococcal infection studies. In all experiments, mice were inoculated intravenously (i.v.) into the tail vein with 0.2 ml of staphylococcal suspension. At the end of experiments, the mice were anaesthetized with ketamine hydrochloride (Pfizer AB, Sweden) and metedomidine (Orion Pharma, Finland) before blood from the axillary artery was collected. Afterwards the mice were immediately sacrificed by a cervical dislocation.

In the first three experiments, the effect of IL-1Ra on staphylococcal arthritis was assessed. The mice (n = 10 mice/group for each experiment) were inoculated with an arthritogenic dose of 1.1–1.7x10^6^ cfu of *S*. *aureus* strain Newman. The mice were regularly weighed and clinically examined for arthritis incidence and severity by two observers (T.J. and A.A.) blinded to the treatment groups. After sacrificing the mice at day 10, the kidneys were obtained for the assessment of bacterial persistence, serum samples were collected to assess the cytokine levels, and the paws were obtained for radiological examination of bone erosions. Thereafter, the paws were further microscopically evaluated for the expression of synovitis and destruction of cartilage and bone.

One experiment was carried out to assess the effect of IL-1Ra treatment on staphylococcal sepsis. The mice (n = 10 mice/group) were inoculated with a septic dose of 2.2x10^7^ cfu of *S*. *aureus* strain Newman. The survival of mice was examined by the observers (T.J. and A.A.) blinded to the treatment groups every 12 hours. When a mouse was judged too ill to survive until the next time point, it was sacrificed and considered dead due to sepsis [20].

To study the effect of IL-1Ra treatment on bacterial load locally in the joints, mice pretreated with IL-1Ra or PBS (n = 10/group) were inoculated with *S*. *aureus* Newman strain (1.7x10^6^ cfu/mouse). The joints (wrists, ankles, and knees) that are most often affected by *S*. *aureus* were collected and homogenized for cfu counts on day 5 when the clinical arthritis difference became evident.

### Treatment with IL-1 receptor antagonist

Anakinra (Kineret; Swedish Orphan Biovitrum AB) was used for the IL-1Ra treatment and has been previously shown to block biological function of murine IL-1 [21]. Anakinra (0.4mg/mouse in 0.1 mL of PBS) was given subcutaneously daily for seven days before inoculation of the mice with *S*. *aureus* and the treatment was continued for 10 days post-infection.

### Clinical evaluation of arthritis

Observers (T.J. and A.A.) blinded to the treatment groups visually inspected all 4 limbs of each mouse. Arthritis was defined as erythema and/or swelling of the joints. To evaluate the severity of arthritis, a clinical scoring system ranging from 0–3 was used for each paw (0 –no inflammation; 1- mild visible swelling and/or erythema; 2- moderate swelling and/or erythema; 3-marked swelling and/or erythema). The arthritis index was constructed by adding the scores from all 4 limbs for each animal as described previously [22, 23].

### Bacteriologic examination

The kidneys from the mice were aseptically removed and blindly assessed by two investigators (T.J. and A.A) for abscesses. A scoring system ranging from 0–3 was used (0- healthy kidneys; 1–1 to 2 small abscesses in the kidneys without structure changes; 2- more than 2 abscesses, but < 75% kidney tissue involved; and 3- large amounts of abscess with >75% kidney tissue involved). The kidneys or joints were homogenized, diluted serially in PBS, and transferred to agar plates containing 5% horse blood. Bacteria were grown for 24 hours and quantified as colony forming units (CFUs).

### Microcomputed tomography (micro-CT)

The joints were fixed in 4% formaldehyde for 3 days and then transferred into PBS for 24 hours. Afterwards all joints from 4 limbs were scanned and reconstructed into a three-dimensional structure with Skyscan1176 micro-CT (Bruker, Antwerp, Belgium) with a voxel size of 35 μm. The scanning was done at 55kV/455 mA, with a 0.2 mm aluminium filter. Exposure time was 47 ms. The X-ray projections were obtained at 0.7° intervals with a scanning angular rotation of 180°. The projection images were reconstructed into three-dimensional images using NRECON software (version 1.5.1; Bruker). After reconstruction, the 3D structures were assessed by two observers (T.J. and A.A.) using CTVox software in a blinded manner. A scoring system ranging from 0–3 (1- mild bone erosion; 2- moderate bone erosion; 3- marked bone erosion) was used[24].

### Histopathological examination of joints

After the scanning, the joints were decalcified, embedded in paraffin and sectioned with microtome. Tissue sections were thereafter stained with haematoxylin and eosin. All the slides were coded and assessed under microscope in a blinded manner by the observers (T.J and A.A) with regard to the degree of synovitis and cartilage and bone destruction. The extent of synovitis and cartilage bone destruction was judged on a scale from grade 0 to 3 as previously described [23, 24].

### Whole blood killing assay

Mice were pretreated with anakinra (n = 5) or PBS (n = 5) for one week as described above. Mouse whole blood samples were collected into heparin-containing tubes and peritoneal leukocytes were collected using peritoneal lavage with 5 ml ice cold PBS. Blood samples were treated with anakinra (0.02mg/ml) for 30’ before the whole blood killing assay.

The impact of IL-1Ra on the bacterial killing capacity of mouse blood was tested by incubating the *S*. *aureus* Newman strain with blood from both IL-1Ra treated mice and controls. Bacterial suspensions were prepared and added into the blood to a final concentration of approximately 1×10^3^ CFU/ml. The mixtures were shaken (300 rpm) at 37°C for 2 hours. To determine bacterial viability, aliquots were withdrawn at the beginning of the assay and after 0.5 and 2 hours of incubation and serial dilutions were plated onto horse blood agar plates.

### Peritoneal macrophage phagocytosis assay

An imaging flow cytometry-based method (ImageStreamX MkII, Amnis) was employed to analyze phagocytic capacity of intraperitoneal macrophages, as previously described [19]. Briefly, peritoneal leukocytes treated with anakinra (0.02 mg/ml) for 1h were allowed to interact with GFP-expressing *S*. *aureus* (MOI 5) at 37°C for 30 min. The cells were fixed in 2% paraformaldehyde, unspecific binding was blocked using Fc-block (BioLegend), and macrophages were stained with PE-Cy7-conjugated rat anti-mouse F4/80 antibody (eBioscience). The internalization wizard in the IDEAS analysis software (v. 6.1) was used to determine whether the GFP-positive bacteria had been internalized by or merely bound to the macrophages. The internalization wizard first identifies cells that are positive for the phagocytic prey, and then discriminates between cells where the image of the phagocytic prey overlaps with the image of the cell (internalized) or not (associated), where the cell boundaries are defined by the surface staining (F4/80)[25]. Data are presented as the percentage of macrophages with associated or internalized *S*. *aureus*.

### Measurement of cytokine levels

The levels of TNF-α, IL-6, IL-4, IL-17, IFNγ, IL-10 and IL-2 in serum were determined using Cytometric Bead Array (CBA) Mouse Th1/Th2/Th17 Cytokine Kit (BD Biosciences) and analysed using the FacsCanto2 flow cytometer. The data were analysed using the FCAP array software (BD Biosciences). The level of RANKL in serum was quantified using DuoSet ELISA Development System Kits (R&D Systems, Abingdon, UK) according to the manufacturer's protocols.

### Statistical analysis

The statistical significance between groups was assessed using the Mann–Whitney *U* test and the χ2 test. The survival of mice was assessed using Kaplan-Meier survival curve analysis. The GraphPad Prism (version 6) software was used for calculations. The results are reported as the mean ± the standard error of the mean (SEM) or median. A two-tailed p value of <0.05 was considered statistically significant.

## Results

### Both severity and frequency of clinical arthritis were significantly increased in IL-1Ra treated mice

**Fig 1:**
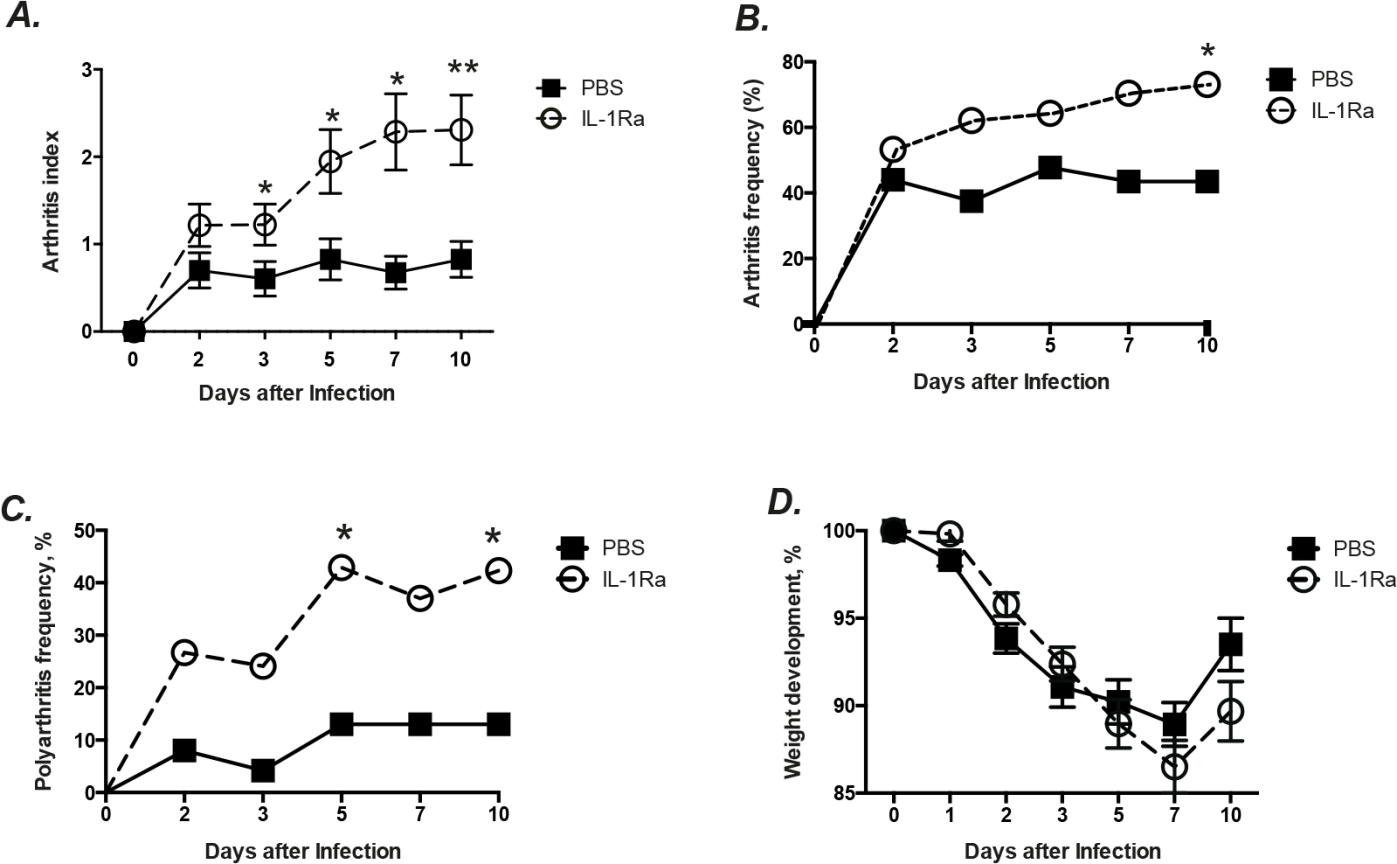
IL-1Ra therapy aggravated *S*. *aureus* arthritis in mice. NMRI mice inoculated with *S*. *aureus* strain Newman (1.1–1.7 × 10^6^ cfu/mouse) were treated with anakinra (IL-1Ra, 0.4mg/mouse, n = 30), or PBS (n = 25) daily starting on day 7 before inoculation with bacteria and continuing until the animals were sacrificed on day 10. The severity (A) and frequency (B) of clinical arthritis in the mice were observed for 10 days post-infection. The frequency of polyarthritis (C) and changes in percentage of body weight (D) of the mice were also registered. The data from 3 independent experiments were pooled. Statistical evaluations were performed using the Mann–Whitney *U* test and chi-square test. Data are presented as mean±SEM. * = *p* < 0.05; ** = *p* < 0.01.

IL-1Ra therapy significantly increased the severity of arthritis in the mice compared to the PBS treated controls. The difference between the groups was observed already on day 3 after bacterial inoculation (p = 0.04) and by day 10 the difference between the groups was even more apparent (p = 0.009, Fig 1A).

Not only was the severity of arthritis higher in the IL-1 Ra treatment group, but also the frequency of arthritis was significantly increased. On day 3, sixty-two percent of the mice in IL-1Ra treated mice developed arthritis, whereas the arthritis frequency in the controls was 38% (Fig 1B). At the end of experiment (day 10), the IL-1 Ra treated group had an arthritis frequency of 73% as compared to 44% of the PBS treated controls (p = 0.04).

### IL-1 Ra therapy significantly increases polyarthritis frequency in mice

IL-1Ra therapy led the mice to develop significantly more polyarthritis, which is indicative of poor prognosis of the disease in septic arthritis [26]. Twenty seven percent of the IL-1Ra treated mice developed polyarthritis already on day 2 compared to 8% of the PBS controls (p = 0.09, Fig 1C). By day 5, 43 percent of IL-1 Ra treated mice developed polyarthritis, whereas for the PBS treated controls the polyarthritis frequency was significantly lower, only 13% (p = 0.03) and this difference remained unchanged until day 10.

### Mice receiving IL-1Ra had similar weight development as the controls

More severe weight loss is usually associated with the worse outcome in our mouse model for septic arthritis [23]. However, we found no difference between the groups regarding the weight loss. IL-1Ra treated mice lost at most 12% of their total body weight by day 7 but recovered to around 91% of their initial total body weight by day 10. The PBS treated control mice lost 10% at most and recovered up to 95% of their body weight at the end of the experiment (Fig 1D). During the course of septic arthritis, 4 mice in IL-1Ra group and 2 mice in PBS group died (ns).

### IL-1Ra treated mice had more severe radiological signs of bone destruction

**Fig 2:**
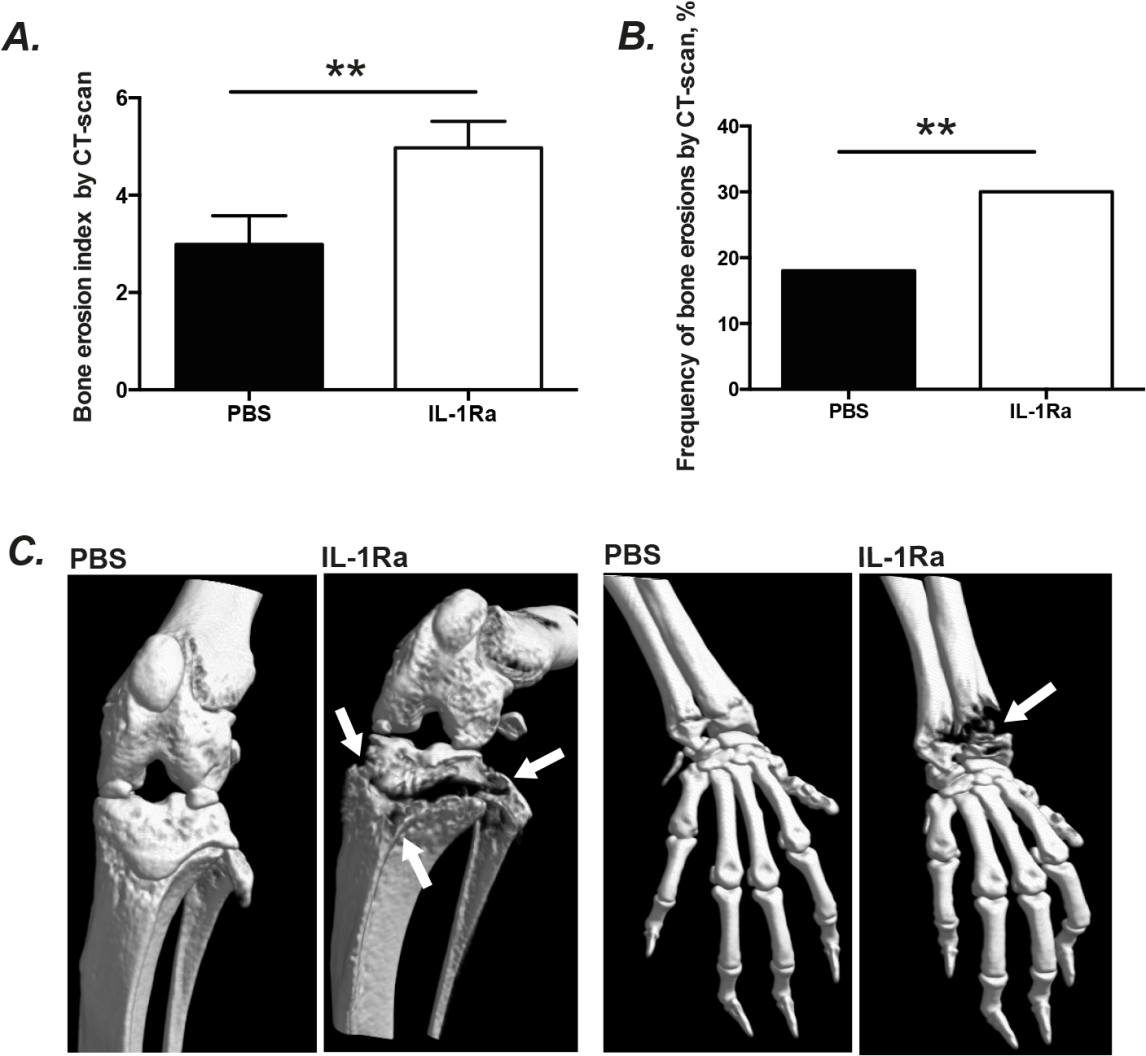
IL-1Ra therapy led to more severe radiologically verified bone destruction in mice. NMRI mice inoculated with *S*. *aureus* strain Newman (1.1–1.7 × 10^6^ cfu/mouse) were treated with anakinra (IL-1Ra, 4mg/mouse, n=30), or PBS (n= 25) daily starting on day 7 before inoculation with bacteria and continuing until the animals were sacrificed on day 10. The accumulative bone destruction scores (A) and frequency of bone destructions (B) from all 4 limbs of NMRI mice by a microCT-scan. Representative CT-scan pictures (C) show intact wrist and knee joints from a NMRI mouse inoculated with *S*. *aureus* Newman strain that was treated with PBS and a heavily destructed knee (proximal tibia and head of fibula) and wrist (both distal radius and ulna) joints from a NMRI mice with septic arthritis treated with anakinra. Arrows indicate the bone destructions. Statistical evaluations were performed using the Mann–Whitney *U* test. Data were presented as mean± SEM. ** = *p* < 0.01; μCT= Micro Computed Tomography.

To confirm our clinical arthritis data, the state of the art technique, micro-CT (μCT) was applied to determine whether IL-1Ra treatment led to increased bone destruction. Importantly, μ-CT allows us to judge the destruction of bones where clinical arthritis scoring is impossible to assess, such as the knee and elbow joints. We found that both the severity (p = 0.005) and frequency (30% vs. 18%, p = 0.009) of bone erosion were significantly higher in the IL-1 Ra treated mice as compared to the PBS treated controls (Fig 2A–2C).

Furthermore, a more detailed sub-group analysis was performed to investigate which particular joints were affected by *S*. *aureus* infection in mice receiving IL-1Ra. Both the severity and frequency of bone destruction were significantly higher in the hands (i.e. wrists and the fingers) of the IL-1Ra treated mice compared to the control mice (Table 1). The feet (including ankles and toes) of IL-1Ra treated mice exhibited significantly severe bone destruction compared to the PBS treated mice (p = 0.03). Additionally, 44% of all knee joints of mice receiving IL-1Ra displayed the signs of bone destruction in comparison with 26% of knees of control animals (p = 0.06). In contrast, no difference was found in elbow joints between groups. Our data suggest that IL-1Ra treatment facilitates bacteria to invade certain joints including joints in hands and feet, but not elbow joints.

**Table 1:** Subgroup analysis of bone destructions by a μCT-scan.

	Severity (Mean ± SEM)		Frequency (%)	
	PBS (n = 46)	IL-1 Ra (n = 52)	PBS (n = 46)	IL-1Ra (n = 52)
Hands	0.7 ± 0.2	1.7 ± 0.3 *	17	40 *
Elbows	0.2 ± 0.1	0.2 ± 0.1	9	6
Knees	1.4 ± 0.3	2.2 ± 0.4	26	44
Feet	0.6 ± 0.1	1.0 ± 0.2 *	23	29

Statistical evaluations were performed using the Mann–Whitney *U* test and chi-square test. Data were presented as mean±SEM.

* = *p* < 0.05.

### Mice receiving IL-1Ra therapy displayed more histological signs of septic arthritis

**Fig 3:**
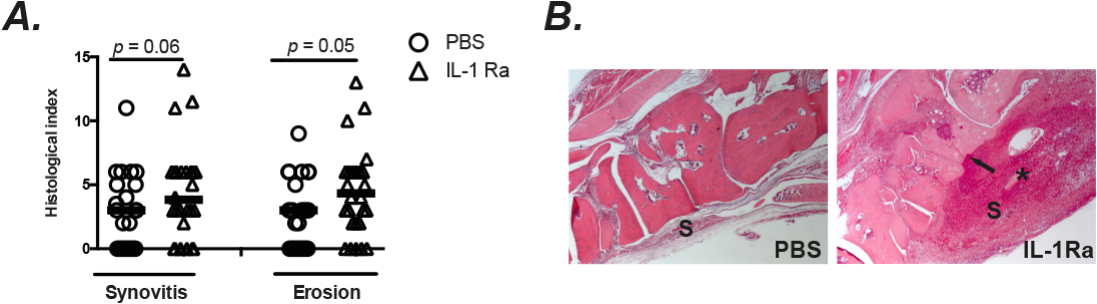
IL-1Ra treated mice had more histological signs of septic arthritis. NMRI mice inoculated with *S*. *aureus* Newman strain (1.1–1.7 × 10^6^ cfu/mouse) were treated with anakinra (IL-1 Ra 4mg/mouse, n = 30), or PBS (n = 25) every day starting on day 7 before inoculation with bacteria and continuing until the animals were sacrificed on day 10. (A) Histological evaluation (including the synovitis scores and bone erosion scores) of the joints from all 4 limbs on 10 days after infection. (B) Left panel: A micrograph of histologically intact midfoot joints from a NMRI mouse inoculated with *S*. *aureus* strain Newman that was treated with PBS. Right panel: A micrograph of a heavily inflamed midfoot joints with bone and cartilage destruction from a NMRI mouse with septic arthritis treated with anakinra. Hematoxylin/eosin was used for staining; * indicates heavely inflamed synovium; Arrow indicates the bone erosion; S, synovial tissue. Statistical evaluations were performed using the Mann–Whitney *U* test. Data were presented as scatter dot plot with median; ns = not significant.

In line with results from both clinical and radiological signs of septic arthritis, the histopathological changes of septic arthritis also tended to be more severe in mice receiving IL-1Ra compared to control mice. The extent of joint destruction as well as the histologically verified synovitis were enhanced in IL-1Ra treated mice compared to the PBS treated mice (Fig 3A and 3B).

### Bacterial clearance was not affected by IL-1Ra therapy

**Fig 4:**
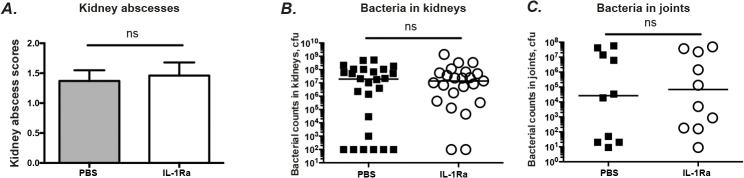
IL-1Ra therapy has no effect on either kidney abscesses or *S*. *aureus* loads in kidneys and joints. NMRI mice inoculated with *S*. *aureus* strain Newman (1.1–1.7 × 10^6^ cfu/mouse) were treated with anakinra (IL-1Ra, 4mg/mouse, n = 40), or PBS (n = 35) every day starting on day 7 before inoculation with bacteria and continuing until the animals were sacrificed on day 5 and day 10. (A) Abscess scores of the kidneys from the mice sacrificed 10 days after infection and, (B) persistence of *S*. *aureus* in kidneys of the mice. The data from 3 independent experiments were pooled, (n = 23–26 mice/group). (C) Persistence of *S*. *aureus* in joints including wrists, ankles, and knees of the mice (n = 10 mice/group) sacrificed 5 days after infection. Statistical evaluations were performed using the Mann–Whitney *U* test. Data were presented as mean± SEM for kidney abscesses or scatter dot plot with median for bacterial load in kidneys and in joints. ns = not significant.

No significant difference was found between IL-1Ra-treated mice and the PBS-treated controls regarding kidney abscess scores and the actual bacterial count in the kidneys (Fig 3A and 3B). Similarly, no difference was found regarding the percentage of negative kidney counts between IL-1Ra-treated and PBS-treated groups (23.1% Vs. 8.7%, ns). To elucidate whether deleterious effect on septic arthritis by IL-1Ra is due to poor bacterial clearance in local joints, bacterial counts in the joints were analyzed on day 5 when the clinical arthritis difference became evident (Fig 4C). Joint cfu counts were positive in 90% of animals. Bacterial loads in the joints were similar between IL-1Ra treated mice and PBS group, suggesting that IL-1Ra treatment did not increase the bacterial load locally in the joints.

To assess whether IL-1Ra treatment causes leukopenia, the white blood cell counts were analyzed in the blood from mice treated with anakinra for one week. The white blood cell counts were not altered by IL-1Ra therapy (4.5±0.39x10^9^/L Vs. 5.2±0.59x10^9^/L, ns).

**Fig 5:**
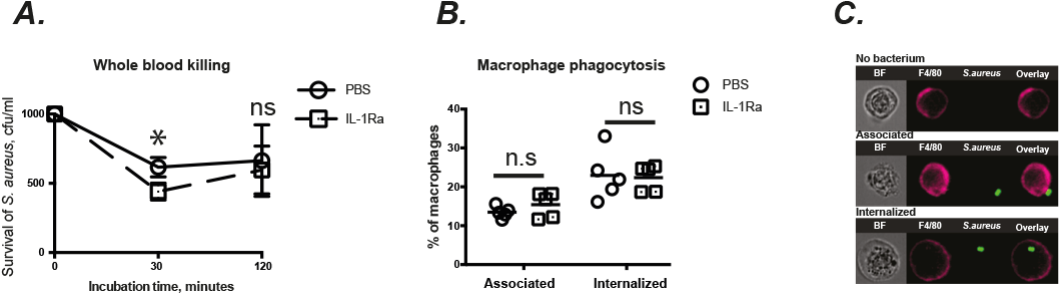
IL-1Ra treatment did not alter whole blood S. aureus killing capacity and macrophage phagocytosis. Blood and peritoneal macrophages were collected from NMRI mice treated subcutaneously with anakinra (IL-1Ra, 4mg/mouse, n = 5), or PBS (n = 5) for 7 days. (A) Survival of *S*. *aureus* Newman strain was analyzed in mouse whole blood, where bacteria were adjusted to 1.0 × 10^3^ CFU/ml, mixed 1:4 with blood, and incubated at 37°C. (B) Quantitative phagocytosis rates and (C) representative micrographs of peritoneal macrophages allowed to interact with GFP-expressing *S*. *aureus* (MOI 5), analyzed by imaging flow cytometry. Statistical evaluations were performed using the Mann–Whitney *U* test. Data were presented as mean± SEM or scatter dot plot with median. * = *p*<0.05; ns = not significant.

After incubation with whole blood for 30’, around 50% of *S*. *aureus* were killed in mice treated with PBS. Surprisingly, a slightly lower number of *S*. *aureus* was found in the blood from IL-1Ra treated mice compared with control mice (*p*<0.05). However, this marginal difference disappeared after 2 hours of incubation (Fig 5A).

In line with the results regarding the bacterial load in the kidneys, no significant difference was found between different treatment groups regarding the leukocyte counts and populations (data not shown) in peritoneal fluid as well as the phagocytic capacity of peritoneal macrophages (Fig 5B and 5C).

### Serum cytokine profiles of mice treated with IL-1Ra

No significant difference was observed regarding the serum levels of RANKL, IL-6, IL-2, IL-4, TNF-α, IL-10, IFN-γ, and IL-17A between the different treatment groups (Table 2).

**Table 2:** Serum levels of cytokines from mice treated with IL-1Ra.

	IL-2 (pg/ml)	IL-4 (pg/ml)	IL-6 (pg/ml)	IL-10 (pg/ml)	IL-17A (pg/ml)	TNF-α (pg/ml)	IFN-γ (pg/ml)	RANKL (pg/ml)
PBS (n = 23)	4.9±0.8	6.1±1.8	109.1±25.8	3.8±1.9	16.9±2.3	21.9±2.8	8.7±1.5	103.7±43.8
IL-1Ra (n = 26)	2.9±0.7	5.9±1.6	122.7±15.1	6.0±2.2	17.7±2.4	23.2±2.2	8.4±1.1	422.9±211.
*p*-value	0.06	0.9	0.09	0.6	0.1	0.4	0.9	0.1

Serum levels of TNF-α, IFN-γ, IL-2, IL-4, IL-6, IL-17A, IL-10, and receptor activator of nuclear factor kappa-B ligand (RANKL) were determined after termination of the experiment on day 10 after infection. Statistical evaluations were performed using the Mann–Whitney *U* test. Data were presented as mean±SEM.

### IL-1Ra therapy increased the mortality of mice with staphylococcal sepsis

**Fig 6:**
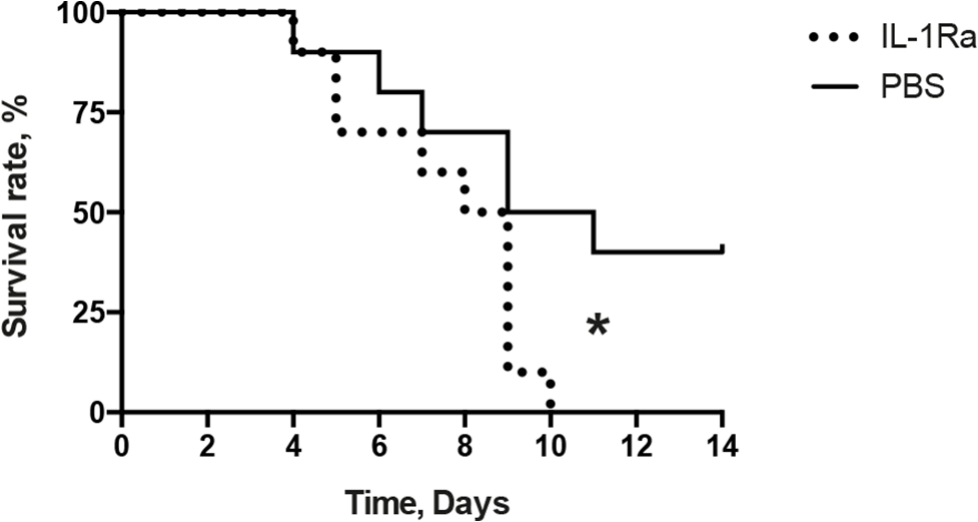
IL-1Ra treatment enhanced the mortality of staphylococcal sepsis. NMRI mice inoculated with a sepsis dose of *S*. *aureus* strain Newman (2.2x10^7^ cfu/mouse) were treated with anakinra (IL-1Ra, 4mg/mouse, n = 10), or PBS (n = 10) every day starting on day 7 before inoculation with bacteria and continuing until the animals were sacrificed on day 14. Statistical evaluations were performed using the log-rank (Mantel-cox) test.

The lethal dose of *S*. *aureus* (2.2x10^7^ cfu/mouse) was used to study the effect of IL-1Ra treatment on staphylococcal sepsis. The course of *S*. *aureus* sepsis were monitored for two weeks. IL-1Ra pre-treatment significantly increased the mortality in staphylococcal sepsis (Fig 6). All the mice from the IL-1Ra treatment group died by day 10 whereas 40% of the control mice survived until the end of experiment on day 14 (p = 0.03).

## Discussion

In this study, we demonstrate that pretreatment with IL-1Ra significantly increased both frequency and severity of clinical arthritis in mice. Importantly, the data of clinical arthritis were in full agreement with results obtained from both μCT-scan and histological evaluation that showed enhanced joint inflammation and bone erosions in IL-Ra treated mice. Also, polyarthritis, a more severe form of septic arthritis [15, 27], was more frequently found in mice receiving IL-1Ra treatment. Polyarthritis is known to be associated with higher mortality in septic arthritis patients compared to patients with septic monoarthritis [27]. Indeed, mice receiving IL-Ra had higher mortality than control mice in our experimental setting.

Knee joints are the most common joints that are engaged in septic arthritis in humans [15], which is actually in line with our data in mice- 26% of all knee joints compared with only 9% of elbows were affected. Intriguingly, μCT-scan analysis revealed that IL-1 Ra treatment led to significantly more severe and frequent septic arthritis in smaller joints e.g. joints in hands that are usually not so commonly affected by *S*. *aureus* in humans. There was also a clear trend that more knee joints were engaged in septic arthritis in IL-1Ra treatment group. However, both frequency and severity of septic arthritis in elbow joints remained unchanged in mice receiving IL-1Ra, suggesting that the detrimental effect of IL-1Ra treatment in septic arthritis is limited to certain joints e.g. wrists, ankles, knees, but not elbow joints.

Tarkowski, et al showed that IL-1R-/- mice developed more severe septic arthritis than wild type mice [13]. IL-1R deficiency also significantly increased the mortality of the mice compared to wild type mice, which is in agreement with our present study. In that study bacterial load in the kidneys was significantly higher in IL-1R-/- mice compared with WT mice, whereas in our study, both the kidney abscesses score and bacterial load in the kidneys were similar in IL-1 Ra treated animals and control mice. This suggests that blocking biological activity of IL-1 by IL-1Ra might not result in the same clinical outcome e.g. kidney abscesses as total absence of the IL-1 receptor.

The definitive criterion for diagnosis of septic arthritis is the direct demonstration of bacteria in the joint fluids [28]. The fact that the number of joints affected by septic arthritis was significantly higher in IL-1Ra treated mice suggests that IL-1Ra treatment facilitates the bacterial invasion into the joints. Neutrophils are known to play the vital protective role in *S*. *aureus* infections and mice receiving granulocyte-depleting antibodies are more susceptible to septic arthritis compared with control animals [29]. Accumulation of neutrophils in both lungs and skin were greatly enhanced by local administration of IL-1 [30, 31], whereas IL-1Ra inhibited IL-1-induced neutrophilic infiltration in lungs [32]. In a *Pseudomonas aeruginosa* pneumonia model, neutrophil influx in bronchoalveolar lavage was significantly decreased in IL-1R-/- mice and in control mice treated with IL-1 Ra [33]. Abscess formation is also considered as a potent innate immune response to infection, which prevents the systemic bacterial dissemination. Indeed, IL-1β produced by neutrophils has been shown to be crucial for abscess formation in a mouse model of *S*. *aureus* cutaneous infection [34]. Also, treatment with IL-1Ra increased the bacterial burden in the lungs in a rabbit model of pneumonia [35]. Thus, different lines of evidence suggest the hypothesis that IL-1Ra might hamper the innate protective function of neutrophils. However, we did not observe any deteriorated bacterial killing capacity by whole blood from mice receiving IL-1Ra. Indeed, unaltered bacterial load in the kidneys by IL-1Ra also suggest the innate immune defense in the blood stream is probably unaffected by IL-1Ra therapy.

Another potent player to defend the host from *S*. *aureus* invasion into local joints is macrophages. At the initial phase of infection, monocytes are effectively recruited to the inflammation site, being stimulated by cytokines such as IFNγ and IL-4 to differentiate into macrophages. IL-1β was shown to prevent the apoptosis of monocytes at the inflammation site and prolonged the viability of monocytes as well as directing their differentiation towards proinflammatory macrophages [36]. Thus treatment with IL-1Ra might reduce the number of proinflammatory effector macrophages, leading to diminished host immune defense and more joint infections. Serum levels of IL-2, a Th1 cytokine, tended to be decreased in IL-1Ra treated mice, which indeed reflect the down-regulation of Th1 response and consequent inactivation of macrophages. However, in our experimental setting the phagocytic capacity of peritoneal macrophages was not altered by IL-1Ra treatment. Also, bacterial counts in local joints were not affected by IL-1Ra treatment. Thus, the mechanism responsible for our observed phenomenon still remains elusive and needs to be addressed in further studies.

Is this murine study relevant for humans in a clinical setting? Because of the different pharmacokinetics in human and mouse, the doses of anakinra (IL-1Ra) administered to the mice in this study might not be directly comparable with the doses used in patients. Also, in present study, healthy mice without any previous underlying diseases were used, whereas in a clinical situation the patients receiving IL-1Ra therapy usually have underlying rheumatic disorders. Therefore, the clinical significance of our findings should be interpreted with care while translating the results from mice to man. However, one of the most common symptoms of autoinflammatory syndromes is joint inflammation [2], and preexisting joint disease is known to be one of the risk factors for acquiring septic arthritis [15]. One can speculate that compared with IL-1Ra treated healthy mice, patients receiving IL-1Ra therapy might have a further enhanced risk of acquiring infections in their already inflamed joints.

Our data demonstrate that mice receiving IL-1Ra treatment not only have an increased risk of *S*. *aureus* septic arthritis, but also worse prognosis once they develop septic arthritis. If our findings are also valid in the human setting, patients with high risk of *S*. *aureus* bacteremia e.g. undergoing hemodialysis or peritoneal dialysis [37] might be considered to refrain from IL-1Ra treatment to avoid severe destructive septic arthritis or life-threatening *S*. *aureus* sepsis. As for patients already undergoing IL-1Ra therapy, careful monitoring for signs of infection is required. Indeed, it is strongly recommended to discontinue immunosuppressive treatments including IL-1Ra treatment if there are apparent signs of systemic infections in patients. Since the half-life of anakinra is relatively short, immediate discontinuation of IL-Ra treatment might decrease the risk of further aggravation of the infection.
